# Activation of RAGE leads to the release of glutamate from astrocytes and stimulates calcium signal in neurons

**DOI:** 10.1002/jcp.30324

**Published:** 2021-02-11

**Authors:** Anna Kamynina, Noemi Esteras, Dmitry O. Koroev, Plamena R. Angelova, Olga M. Volpina, Andrey Y. Abramov

**Affiliations:** ^1^ Research Center for Molecular Mechanisms of Aging and Age Related Diseases Moscow Institute of Physics and Technology (National Research University) Dolgoprudny Russia; ^2^ Shemyakin‐Ovchinnikov Institute of Bioorganic Chemistry RAS Moscow Russia; ^3^ Department of Clinical and Movement Neurosciences UCL Queen Square Institute of Neurology, Queen Square London UK

**Keywords:** astrocytes, calcium signal, glutamate, neurons, RAGE, VGLUT

## Abstract

The receptor for advanced glycation end products (RAGE) is a signal receptor first shown to be activated by advanced glycation end products, but also by a variety of signal molecules, including pathological advanced oxidation protein products and β‐amyloid. However, most of the RAGE activators have multiple intracellular targets, making it difficult to unravel the exact pathway of RAGE activation. Here, we show that the cell‐impermeable RAGE fragment sequence (60–76) of the V‐domain of the receptor is able to activate RAGE present on the plasma membrane of neurons and, preferentially, astrocytes. This leads to the exocytosis of vesicular glutamate transporter vesicles and the release of glutamate from astrocytes, which stimulate NMDA and AMPA/kainate receptors, resulting in calcium signals predominantly in neurons. Thus, we show a specific mechanism of RAGE activation by the RAGE fragment and propose a mechanism by which RAGE activation can contribute to the neuronal‐astrocytic communication in physiology and pathology.

## Graphical abstract

The cell‐impermeable receptor for advanced glycation end products (RAGE) fragment is able to activate RAGE, which presented on the plasma membrane of neurons and, preferentially, astrocytes. This leads to the exocytosis of VGLUT2 vesicles and the release of glutamate from astrocytes, which stimulate NMDA and AMPA/kainate receptors, resulting in calcium signals predominantly in neurons.

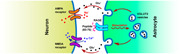

**Abbreviations**
AD: Alzheimer's disease
[Ca^2+^]_c_
: cytosolic calcium concentration
RAGE: receptor for advanced glycation end‐products
SERCA: SarcoEndoplasmic Reticulum Ca^2+^‐ATPase
V‐ATPase: vacuolar type H^+^ ATPase
VGCC: voltage‐gated calcium channel

## 1 INTRODUCTION

The receptor for advanced glycation end products (RAGE) is a signal transduction receptor in the form of a transmembrane protein that belongs to the immunoglobulin superfamily. RAGE is a pattern recognition receptor and is able to sense a number of signal molecules, including advanced glycation end products (Neeper et al., 1992), β‐amyloid, phosphatidylserine, advanced oxidation protein products, S100 proteins, and others (Fritz, 2011). It is believed that RAGE is highly upregulated in the time of development of various diseases, such as diabetes, cardiovascular diseases, cancer, and neurodegeneration (Bongarzone et al., 2017). Considering this, in the last decade, several therapeutic strategies have been developed to block RAGE for the treatment of these diseases (Bongarzone et al., 2017; Chhip et al., 2019). However, there is limited information about the mechanism of RAGE signaling and a growing interest in unraveling the intracellular pathways by which RAGE controls these physiological and disease‐related processes.

Expression of RAGE has been described in neurons, astrocytes, and microglia from different brain areas. Although RAGE is shown to be involved in the development of Parkinson's disease (Viana et al., 2016), Alzheimer's disease, stroke, and neuroinflammation, an important role for this receptor in physiological processes has been also demonstrated. Thus, vascular RAGE transports oxytocin into the brain to elicit maternal bonding behavior in mice (Yamamoto et al., 2019). RAGE also regulates a number of cell processes of pivotal importance, such as proliferation, neuronal differentiation, and autophagy (Kim et al., 2012). Activation of RAGE by its ligands induces the release of cytokines, interleukins, and increased reactive oxygen production in glial cells. Importantly, RAGE interacts with the Ca^2+^‐modulated protein, S100B, which can also be released from astrocytes and modify calcium signal (Donato et al., 2013).

Synthetic RAGE fragments have been used in various experiments to unravel the mechanism of receptor activation/inhibition and for the development of a cell‐protective strategy. Thus, the synthetic fragment (60–76) protects the spatial memory of mice with an experimentally induced form of Alzheimer's disease and lowers the level of brain β‐amyloid in experimental animals (Volpina et al., 2015, 2018). Recently, we have shown that various short RAGE fragments could protect primary cultures of neurons and astrocytes against β‐amyloid toxicity by binding β‐amyloid (Kamynina et al., 2018). RAGE is a transmembrane protein composed of three major parts: an extracellular region with one V‐domain and two C‐domains, a transmembrane region, and an intracellular tail (Neeper et al., 1992). The V‐domain is responsible for interaction with multiple RAGE ligands. One of the most active RAGE peptide fragments (sequence 60–76) is from the V‐domain, and we have suggested that the effect of this peptide could not be explained only by binding to a potential RAGE ligand, such as β‐amyloid, but also by the interaction of this fragment with RAGE.

Here, we studied the effect of this RAGE fragment on the calcium homeostasis of primary neurons and astrocytes. We have found that the RAGE fragment (sequence 60–76) induces calcium signals in neurons through the activation of RAGE on the membrane of astrocytes, which leads to the release of glutamate from vesicular glutamate transporter (VGLUT) vesicles and activates glutamate‐induced calcium signal in neurons. Thus, RAGE activation can contribute to the neuronal‐astrocytic communication in physiology and pathology.

## 2 MATERIALS AND METHODS

### 2.1 Peptide synthesis

Peptide fragment (60–76) of RAGE with the sequence AWKVLSPQGGGPWDSVA and peptide fragment (60–62) with the sequence AWK were derived from the human RAGE (Q15109 UniProtKB/SwissProt). In Flu‐peptide, fluorescein was coupled to the N‐terminus of the protected peptide (60–76) attached to the resin via an aminocaproic acid spacer. For purification of rabbit antibodies peptide (60–76) with two additional Lys residues – K‐Ahx‐(60–76)‐K in which N‐terminal Lys residue was attached via an aminocaproic acid spacer (Ahx) (Lys_2_‐peptide), was used.

Peptides were synthesized by the solid‐phase method and purified as described previously (Volpina et al., 2018).

### 2.2 Preparation of affinity‐purified antibodies against (60–76) fragment

#### 2.2.1 Peptide conjugates with keyhole limpet hemocyanin (KLH)

To obtain a conjugate of the peptide (60–76), 1 mg of the peptide was added to 1 ml of the KLH solution in phosphate‐buffered saline (PBS; 5 mg/ml, pH 7.4; Sigma‐Aldrich) and stirred for 30 min. Then, 50 µl of 0.25% glutaraldehyde solution (Sigma‐Aldrich) was added by drops for 1 h. The mixture was incubated for 16 h, and dialyzed against 100‐fold volume excess of PBS for 18 h with a triple change of the buffer.

#### 2.2.2 Immunization

To initiate the production of antibodies against the peptide, two 2–3 kg female rabbits were immunized with KLH conjugate of the peptide. The animal procedures were performed in accordance with the Guide for the Care and Use of Laboratory Animals published by the National Institutes of Health and with the “Regulations for Studies with Experimental Animals” (Decree of the Russian Ministry of Health from the 12th August 1997, No. 755). The protocol was approved by the Institutional Ethics Committee of the Shemyakin‐Ovchinnikov Institute of Bioorganic Chemistry (Protocol No. 162/2015). For immunization, the solution of the conjugate in PBS (2 mg of peptide/ml) was mixed with an equal volume of either Freund's complete adjuvant or Freund's incomplete adjuvant (the 1st or the 2nd immunization, respectively; Disco Laboratories) and the emulsion was then injected subcutaneously into four points along the spinal column at a dose of 1 mg of the peptide per rabbit. Blood samples were collected, and the blood sera were prepared as described in Kamynina et al. (2013). The aliquoted sera samples were stored at −20°C until use.

#### 2.2.3 Experimental schedule of rabbit blood sera collection

First day—the first immunization with KLH‐peptide in Freund's complete adjuvant, 45th day—the second immunization with KLH‐peptide in Freund's incomplete adjuvant, 55th day—blood samples collection.

Affinity adsorbent and affinity chromatography was performed as described in Kamynina et al. (2013). Shortly, 1 g of CNBr‐activated Sepharose 4B (GE Health Care) in 3 ml of 1 mM NaCl was mixed with a solution of 1 mg Lys_2_‐peptide in 1 ml coupling buffer, 0.1 M NaHCO_3_ (pH 8.3). Not bound Lys_2_‐peptide was washed away with the coupling buffer. The remaining active groups were blocked with blocking buffer, 0.2 M Glycine‐NaOH buffer (pH 8.0). The adsorbent was then treated with sequential washes of each buffer with different pH: 0.1 M acetic acid/sodium acetate (pH 4.0) followed by a wash with 0.1 M borate buffer (pH 8.0). The washed adsorbent was stored in the presence of 0.05% NaN_3_ at 4°C until use. One milliliter of sepharose conjugated with Lys_2_‐peptide contains 0.4 mg of the peptide according to amino acid analysis data.

Two milliliters of rabbit blood sera containing antibodies against the peptide (60–76) was applied into the column poured with 5 ml of the Sepharose conjugated with Lys_2_‐peptide. Elution of antibody bound to peptide was done using elution buffer containing 0.1 M glycine–HCl (pH 2.2), and fractions of the eluate were collected into 2 ml Eppendorf tubes containing 0.05–0.1 ml of 1.5 M Tris–HCl (pH 8.7) to prevent antibody aggregation. The protein concentration of the samples was determined using the UV absorbance (280 nm) extinction coefficient and comprised 700 µg/ml. The affinity‐purified antibodies were stored at −20°C until use.

### 2.3 Enzyme‐linked immunosorbent assay (ELISA)

Rabbit blood sera or affinity‐purified antibodies were pooled for analysis by ELISA as described in Udenfriend et al. (1987). In brief, wells of a 96‐well plate Maxisorp (Nunc, Denmark) were coated with 20 μg/ml of the peptide (60–76), incubated with 100 μl prediluted sera or affinity‐purified antibodies starting from dilution 1:40 or 1:1000, followed by the addition of peroxidase‐conjugated goat antibody to rabbit IgG (Sigma). Antibody titers of sera were quantified as ‐lg of an end‐point dilution with OD > 0.1, which three times exceeded the binding with ChromPure rabbit IgG (Johnson ImmunoResearch Laboratories). Antibody titer of sera comprised 5.7, antibody titer of affinity‐purified antibodies comprised 5.4. The working dilution of the purified antibodies was 1:100.

### 2.4 Cell culture

Mixed cultures of hippocampal and cortical neurons and glial cells were prepared as described previously (Vaarmann et al., 2010) with modifications from Sprague‐Dawley rat pups 2–4 days postpartum (UCL breeding colony). Experimental procedures were performed in full compliance with the United Kingdom Animal (Scientific Procedures) Act of 1986 and with the approval of the University College London Animal Ethics Committee. Hippocampi and cortex were removed into ice‐cold PBS (Ca^2+^, Mg^2+^‐free, Invitrogen, Paisley, UK). The tissue was minced and trypsinized (0.25% for 15 min at 37^o^C), triturated, and plated on poly‐d‐lysine‐coated coverslips and cultured in Neurobasal A medium (Invitrogen, Paisley, UK) supplemented with B‐27 (Invitrogen, Paisley, UK) and 2 mM l‐glutamine. Cultures were maintained at 37^o^C in a humidified atmosphere of 5% CO_2_ and 95% air, fed once a week and maintained for a minimum of 12 days before experimental use to ensure expression of glutamate and other receptors. Neurons were easily distinguishable from glia: they appeared phase bright, had smooth rounded somata and distinct processes, and laid just above the focal plane of the glial layer. Cells were used at 12–15 days in vivo (DIV) unless otherwise stated.

### 2.5 RAGE immunostaining

Primary cocultures of neurons and astrocytes were fixed with 4% PFA for 15 min, washed and permeabilized‐blocked for 1 h at room temperature in PBS with 0.2% Triton X‐100% and 10% bovine serum albumin (BSA). Afterward, cells were incubated overnight stepwise at 4°C with 1:500 RAGE mouse monoclonal antibody (Merck #mab5328) and, when indicated, 1:500 glutamine synthetase rabbit antibody (Abcam, ab73593). Afterward, cells were rinsed with 1% BSA in PBS three times and incubated for one hour with the corresponding fluorophore‐conjugated secondary antibodies (antimouse Alexa 647 or antirabbit Alexa 568). When indicated, cells were then counterstained (1:200) with the Alexa Fluor® 488‐conjugated glial fibrillary acidic protein (GFAP) antibody (ab194324) or the Alexa Fluor® 555‐conjugated beta‐III tubulin antibody (ab202519). Cells were then washed three times and Hoechst 10 μM was included in the last wash to stain the nucleus. Coverslips were mounted onto slides with VectaShield Anfifade Mounting Medium. Images were acquired using a Zeiss 710 VIS CLMS confocal microscope equipped with a META detection system and an ×40 (or ×63) oil immersion objective (Zeiss). The 405, 488, 561, and 633 laser lines were used to excite Hoechst, Alexa 488, Alexa 555‐568, and Alexa 647, respectively.

For the visualization of membrane‐located RAGE only, incubation with RAGE primary antibody was done in live cells before the fixation step. Live primary cocultures of neurons and astrocytes were incubated for 40 min at 4°C with the anti‐RAGE mouse monoclonal antibody (1:100 in HEPES‐buffered salt solution [HBSS]), washed, and fixed for 15 min with 4% PFA. The rest of the protocol was similar to the one for intracellular RAGE.

Images are presented as Z‐projections from z‐stacks or single‐plane images. RAGE staining was pseudo‐colored (yellow LUT) for clarity.

### 2.6 Live cell imaging

#### 2.6.1 Calcium measurements

For measurements of [Ca^2+^]_c_ cells were loaded for 30 min at room temperature with 5 μM fura‐2 AM and 0.005% pluronic acid in an HBSS composed of 156 mM NaCl, 3 mM KCl, 2 mM MgSO_4_, 1.25 mM KH_2_PO_4_, 2 mM CaCl_2_, 10 mM glucose, and 10 mM HEPES; pH adjusted to 7.35 with NaOH. In specific experiments, Ca^2+^‐free HBSS with 0.5 mM EGTA was used. Fluorescence measurements were obtained on an epifluorescence inverted microscope equipped with a × 20 fluorite objective. [Ca^2+^]_c_ was monitored in single cells using excitation light provided by a xenon arc lamp, the beam passing a monochromator at 340 and 380 nm (Cairn Research, Kent, UK). Emitted fluorescence light was reflected through a 515‐nm longpass filter to a cooled CCD camera (Retiga; QImaging) and digitized to 12‐bit resolution. All imaging data were collected and analyzed using software from Andor (Belfast, UK). The fura‐2 data has not been calibrated in terms of [Ca^2+^]_c_ because of uncertainty arising from the use of different calibration techniques.

#### 2.6.2 Flu‐peptide visualization

Imaging of the peptide coupled to fluorescein (Flu‐peptide) was performed in a Zeiss 710 confocal laser scanning microscope using a ×40 oil immersion objective. The 488 nm argon laser was used to excite fluorescein, and emitted fluorescence was measured using a bandpass filter from 510 to 560 nm.

#### 2.6.3 Glutamate vesicles exocytosis in astrocytes using VGLUT2–EGFP

For identification of the glutamate compartments in astrocytes, the cultures were transduced with the adenoviral vectors (AVV) AVV–sGFAP–VGLUT2–EGFP. Experiments were performed after 7–10 days of incubation with the virus. sGFAP is a transcriptionally enhanced, bidirectional, shortened GFAP promoter, which drives transgene expression in astrocytes (Figueiredo et al., 2011; Liu et al., 2008). Fusion with EGFP allows for the detection of transgene expression via green fluorescence. The VGLUT2 (NM0534271; P. Angelova et al., 2018) is targeted to glutamatergic vesicles in astrocytes (P. Angelova et al., 2018).

#### 2.6.4 Glutamate release in neurons and astrocytes using iGluSnFR

To monitor glutamate release, primary cocultures of neurons and astrocytes were transfected with the intensity‐based glutamate sensor iGluSnFR (Addgene; Marvin et al., 2013) using Lipofectamine LTX with Plus Reagent (Thermofisher) following the manufacturer's instructions. Forty‐eight hours after transfection, cells were loaded for 30 min with the calcium indicator x‐Rhod‐1 in HBSS. Calcium responses and glutamate release in response to the RAGE fragment were simultaneously measured using a Zeiss 710 LSM confocal microscope with an integrated META detection system. Excitation of iGluSnFR (GFP) and X‐Rhod‐1 were achieved with the 488 and 561 lasers and emitted light was measured at 495–550 and 580–640 nm, respectively (×40 objective). Six independent coverslips were analyzed.

### 2.7 Data analysis and statistics

Data and statistical analysis were performed using OriginPro (OriginLab) software.

## 3 RESULTS

### 3.1 RAGE fragment peptide (60–76) induces Ca^2+^ signals in neurons and astrocytes through activation of RAGE

**Figure 1:**
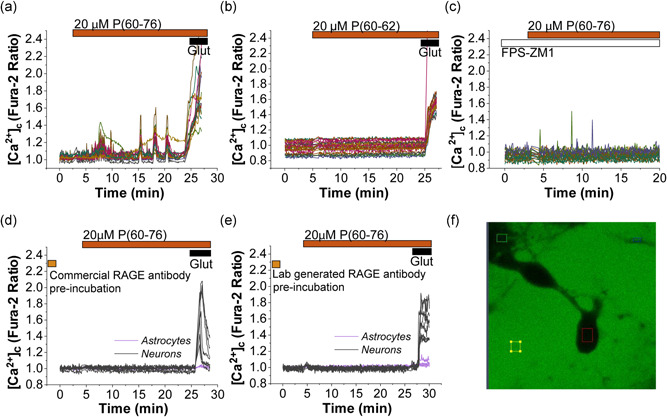
Peptide P (60–76) from the V‐domain of RAGE stimulates calcium signal in neurons through activation of RAGE. Cytosolic calcium changes were monitored using the Fura‐2 ratio in primary cocultures of neurons and astrocytes. Twenty micromolar P (60–76) induced calcium signals in primary neurons (a), while a short version of this peptide used as a control 20 µM P (60–62) had no effect on fura‐2 signal in primary neurons and astrocytes (b). Incubation of the primary coculture of neurons and astrocytes with the RAGE antagonist 1 µM FPS‐ZM1 (10 min; c) or with commercial (d) or generated in the laboratory (e) specific antibodies for the V‐domain of RAGE for 30 min, followed by washing out the antibodies completely inhibited P (60–76)‐induced calcium signal in neurons and astrocytes. Ten micromolar glutamate (Glut) was applied at the end of the experiments for functional neuronal identification. (f) Primary coculture of neurons and astrocytes showed no increase in intracellular fluorescence when incubated with the fluorescein‐conjugated P (60–76) peptide, indicating no penetration of this peptide into the cells. Scale bar = 10 μm. RAGE, receptor for advanced glycation end products

Application of 20 µM peptide (60–76) to primary cultures of neurons and astrocytes induced peak‐like calcium signals in neurons (*n* = 95; Figure 1a) and only in a small proportion of astrocytes (20 of 215 cells). Neurons were easily distinguished from the astrocytes: morphologically by the shape of the soma and functionally as they showed a typical calcium peak in response to 10 µM glutamate (Figure 1a). Importantly, a shorter version of the peptide sequence (60–62), which can be used as a control, did not induce calcium changes either in neurons or in astrocytes (*n* = 3 experiments; Figure 1b).

The effect of the peptide (60–76) was dependent on RAGE activity and could be completely blocked by pre‐incubation of the primary cultures of neurons and astrocytes with FPS‐ZM1 (1 µM,10 min pre‐incubation), a high‐affinity antagonist of RAGE, which binds to the V‐domain of the receptor (*n* = 99 number of cells; Figure 1c). These data suggest that the RAGE peptide (60–76) can bind to the receptor and modulate its activity. To prove this finding, we pretreated the cells for 30 min with two different types of specific antibodies against the V‐domain of RAGE: commercial (Merck #mab5328) and lab‐generated against the 60–76 part of the V‐domain (described in Section 2); and washed them afterward from the solution to prevent interactions. Subsequent application of 20 μM peptide (60–76) did not induce any changes in [Ca^2+^]_c_ of neurons or astrocytes, as depicted in Figure 1d (commercial antibody, *n* = 115 neurons and 110 astrocytes) and Figure 1e (lab‐generated antibody, *n* = 145 neurons and 123 astrocytes). Thus, peptide (60–76) binds to the RAGE receptor and induces calcium signals mostly in neurons.

### 3.2 Peptide 60–76 does not penetrate inside neurons and astrocytes

Peptide (60–76) is water‐soluble and more likely not cell‐permeable. To test this, we used a fluorescein‐labeled peptide (Flu‐peptide) (60–76) to identify if this peptide is able to penetrate through the plasma membrane into the cells. After incubation of the primary coculture of neurons and astrocytes with 20 µM Flu‐peptide (60–76), the cells showed no increase in fluorescence, which was located extracellularly, suggesting that the peptide does not penetrate inside the cells (Figure 1f). Thus, the mechanism by which peptide (60–76) induces calcium signals in the neurons appears to be mediated by the binding of the peptide to RAGE on the plasma membrane.

### 3.3 RAGE is located on the plasma membrane of neurons and astrocytes

**Figure 2:**
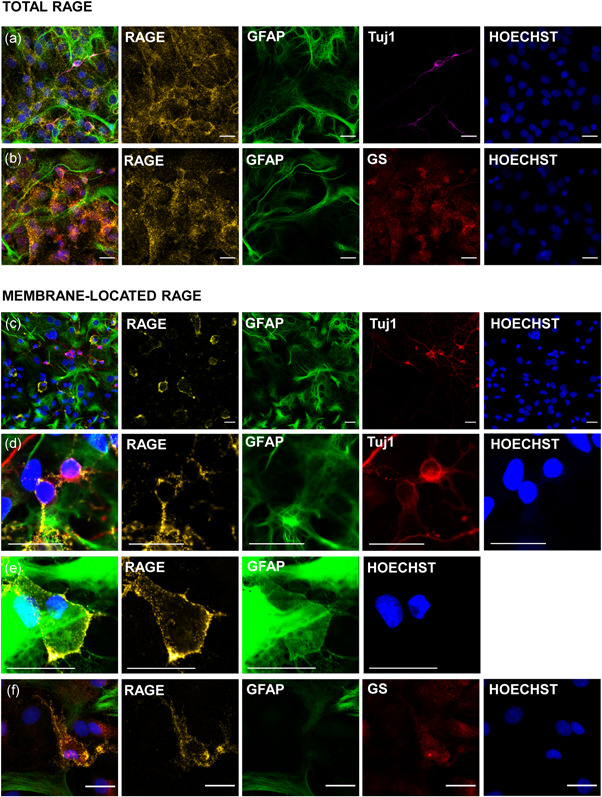
Location of RAGE in primary neurons and astrocytes. Immunostaining of (a, b) total and (c–f) membrane‐located RAGE in primary cocultures of neurons and astrocytes. GFAP and glutamine synthetase (GS) were used as astrocytic markers, Tuj1 (class III beta‐tubulin) as a neuronal marker and Hoechst stained the nucleus. Scale bar = 20 μm. GFAP, glial fibrillary acidic protein; GS, glutamine synthetase; RAGE, receptor for advanced glycation end products

RAGE expression has been described in different cell types in the brain. In our primary cocultures, RAGE is expressed both in neurons and astrocytes, as shown by the colocalization of RAGE immunostaining both with the astrocytic markers GFAP and glutamine synthetase and the neuronal marker Tuj1 (class III‐beta tubulin; Figure 2a,b). To study the expression of membrane‐located RAGE only, live primary cultures were incubated for 40 min at 4°C with the anti‐RAGE antibody before fixation to avoid the internalization of the antibody. As shown in Figure 2c–f, membrane‐located RAGE is expressed in both neurons (Figure 2d) and astrocytes (Figure 2e,f), but preferentially in astrocytes (Figure 2c). Interestingly, in membrane‐RAGE‐positive cells, GFAP staining was weaker than in other astrocytes (Figure 2e). The astrocytic nature of the membrane‐RAGE‐positive cells was proven with an additional astrocytic marker, glutamine synthetase (Figure 2f). Similar results were obtained when substituting the commercial RAGE antibody used in these experiments (Merck, #mab5328) with an anti‐RAGE antibody generated against the fragment sequence (60–76) produced in the laboratory (data not shown). Thus, RAGE is localized on the surface of the plasma membrane of astrocytes and to a lesser degree in neurons in our primary cocultures.

### 3.4 RAGE‐induced calcium signal is dependent on extracellular Ca^2+^ influx

**Figure 3:**
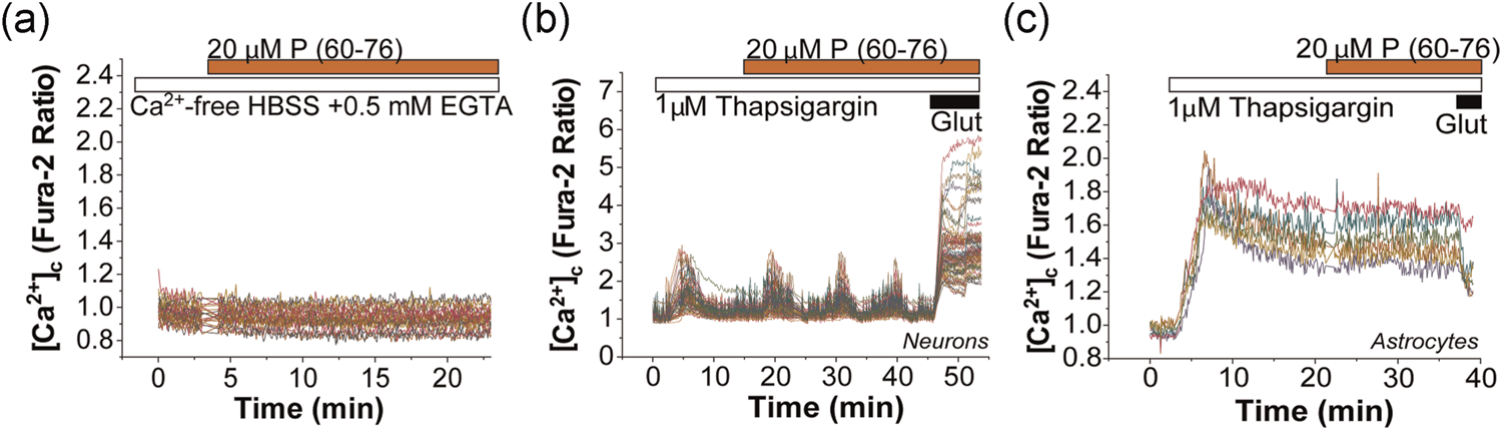
RAGE‐induced calcium signal depends on external Ca^2+^ influx. (a) Incubation of the primary neurons and astrocytes in calcium‐free HBSS (plus 0.5 mM EGTA) completely blocked P (60–76)‐induced [Ca^2+^]_c_ changes. (b, c) Inhibition of the sarco/endoplasmic reticulum Ca^2+^‐ATPase (SERCA) with 1 µM thapsigargin did not prevent RAGE‐induced calcium signal in neurons (b) but blocked it completely in astrocytes (c). Application of glutamate 10 μM at the end of the experiment was used to functionally identify neurons. HBSS, HEPES‐buffered salt solution; RAGE, receptor for advanced glycation end products; SERCA, SarcoEndoplasmic Reticulum Ca^2+^‐ATPase

To identify the source of the calcium signal in neurons in response to the peptide (60–76), we removed the extracellular Ca^2+^ by using calcium‐free HBSS containing 0.5 mM EGTA. We found that removing Ca^2+^ from the media blocks the peptide (60–76)‐induced calcium signal in neurons and astrocytes (*n* = 119 cells Figure 3a; Figure 5c‐d). Thus, the peptide (60–76)‐induced calcium signal is dependent on the external calcium. In addition, to rule out the implication of the intracellular Ca^2+^ stores, we treated the cells with the inhibitor of the sarcoendoplasmic reticulum Ca^2+^‐ATPase (SERCA) thapsigargin (1 µM). The application of thapsigargin empties the Ca^2+^ pool of the endoplasmic reticulum inducing an initial increase in [Ca^2+^]_c_ (Figure 3b,c). We found that subsequent application of peptide (60–76) still induced calcium signals in neurons (Figure 3b; *n* = 189) but not in astrocytes (*n* = 155 astrocytes; Figure 3c). Interestingly, thapsigargin increased the amplitude of the RAGE‐induced calcium signal but not the overall number of cells with signal (Figure 5c,d). Thus, activation of RAGE by peptide (60–76) leads to a Ca^2+^ influx through the plasma membrane of neurons.

**Figure 4:**
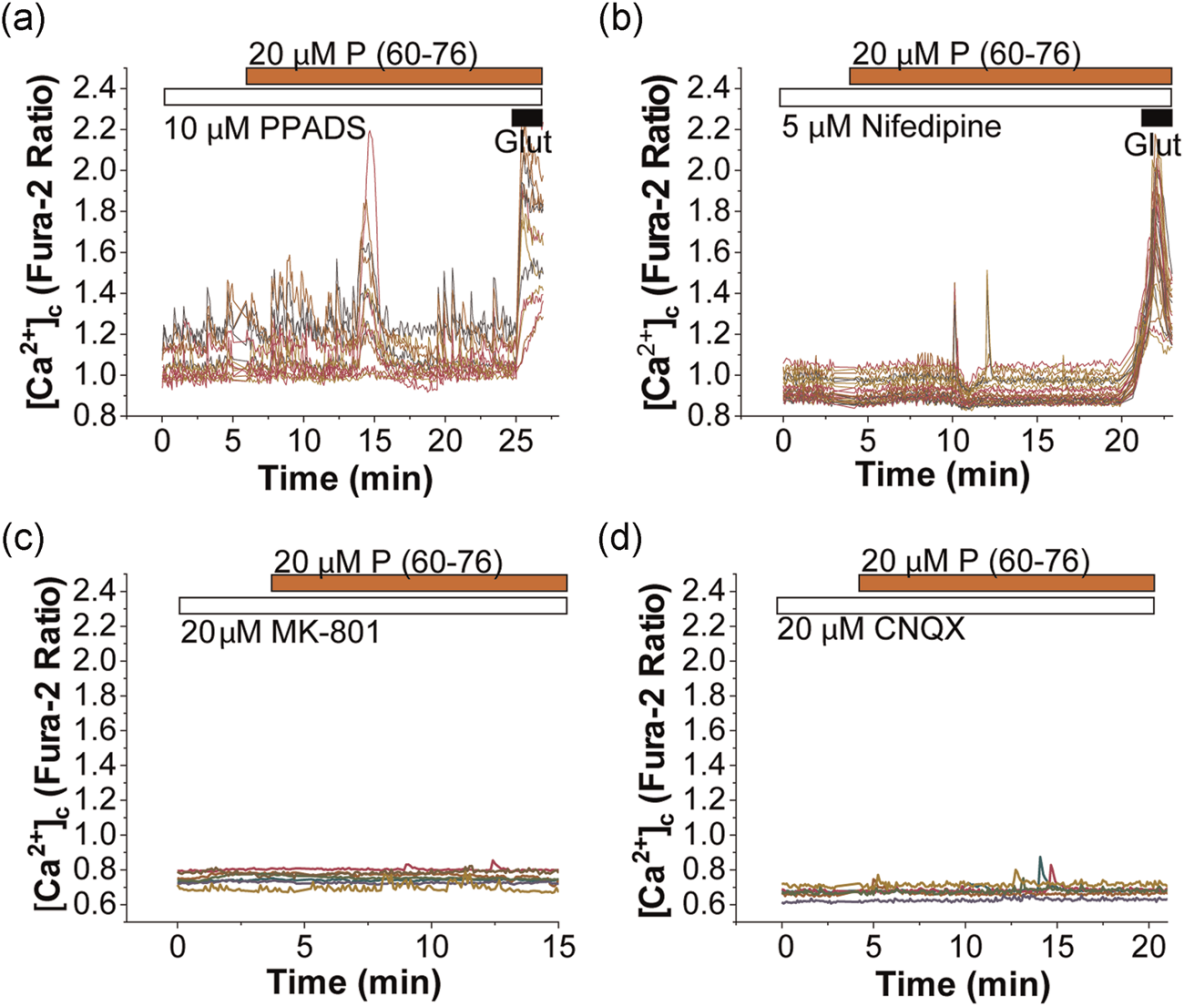
RAGE‐induced calcium signal is mediated by NMDA and AMPA receptors. Primary cocultures of neurons and astrocytes were treated with antagonists of different receptors and channels involved in calcium influx in neurons: (a) P2‐receptor antagonist 20 µM PPADS had no effect on the calcium signal induced by 20 µM P (60–76). (b) Blocker of VGCC nifedipine reduced but did not block P (60–76)‐ induced [Ca^2+^]_c_ changes in neurons. Antagonists of (c) NMDA and (d) AMPA glutamate receptors, 5 µM MK‐801 and 20 µM CNQX, completely blocked the RAGE peptide‐induced calcium signal in neurons. RAGE, receptor for advanced glycation end products

**Figure 5:**
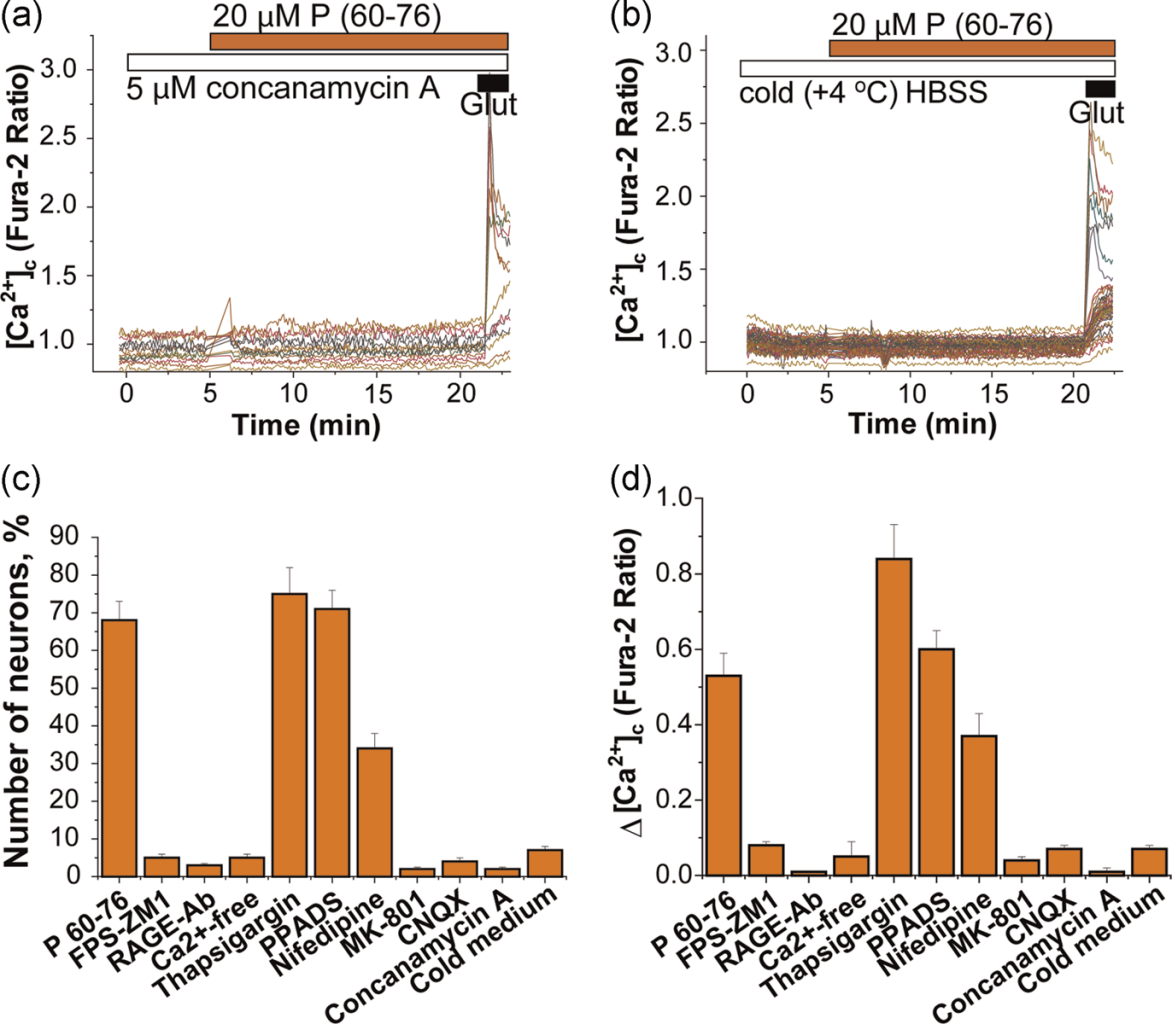
RAGE‐induced calcium signal is dependent on vesicular exocytosis. (a) Primary cultures were pretreated with the inhibitor of V‐ATPases concanamyin A (5 μM) to deplete synaptic vesicles, or incubated in cold HBSS (b) to inhibit temperature‐dependent endo‐exocytosis of vesicles. Both blocked calcium signals induced by peptide P (60–76). The application of glutamate 10 μM at the end of both experiments helped to identify and show that neurons were functional. (c) Percentage of neurons with P (60–76)‐calcium response. (d) Amplitude of the P (60–76)‐calcium response in the fura‐2 ratio. The error bars depict mean ± *SEM*, *p < .05; **p < .01; ***p < .001. HBSS, HEPES‐buffered salt solution; RAGE, receptor for advanced glycation end products

### 3.5 RAGE‐induced calcium signal can be blocked by glutamate receptors antagonists

The influx of external calcium is mediated in neurons by different receptors and channels, including purinergic receptors, voltage‐gated calcium channels (VGCCs), or glutamate receptors (such as the ionotropic NMDA or AMPA receptors). To identify the pathway through which the RAGE peptide induces Ca^2+^ changes in the cells, we inhibited pharmacologically several of these neuronal receptors. Thus, the blocker of purinoreceptors PPADS (20 µM, *n* = 80 Figure 4a) and l‐type VGCC inhibitor nifedipine (5 µM; *n* = 117; Figure 4b) did not abolish the ability of the RAGE peptide to induce Ca^2+^ signals in neurons (Figure 5c,d). However, the antagonists of NMDA (MK‐801 20 µM; *n* = 91) and AMPA/kainate (CNQX 20 µM; *n* = 88) glutamate receptors almost completely blocked peptide (60–76)‐induced calcium signal (Figure 4c,d, see also Figure 5c,d). Thus, the calcium signal in response to the RAGE peptide (60–76) is mediated by glutamate receptors.

### 3.6 RAGE‐induced activation of glutamate receptors can be prevented by inhibition of exocytosis/endocytosis

Glutamate receptors are activated by glutamate released to the extracellular fluid mainly by exocytosis of synaptic vesicles filled with this neurotransmitter. To test whether the activity of the RAGE peptide on Ca^2+^ signal is dependent on vesicular glutamate release, we depleted vesicular glutamate from the ready‐releasable pool by incubating the culture with the specific inhibitor of the vacuolar‐type H^ + ^ATPase (V‐ATPase) concanamycin A (5 µM; 2 h). We found that incubation with concanamycin A prevented the RAGE peptide‐induced elevation of [Ca^2+^]_c_ in neurons (*n* = 99; Figure 5a,c,d), confirming that vesicular glutamate release is an essential mechanism in the manifestation of the RAGE peptide activity.

Importantly, both exocytosis and endocytosis are dependent on temperature and can be blocked by the cold solution. We next applied the RAGE peptide (60–76) to the primary coculture of neurons and astrocytes in HBSS at 4°C (*n* = 88). Incubation of the cocultures in cold solution completely blocked RAGE‐induced calcium signals (Figure 5b), which also confirms that calcium signal in neurons induced by activation of RAGE is due to vesicular (glutamate) release. Note that neurons were functional after incubation with concanamycin A or cold HBSS and showed a calcium peak in response to the application of extracellular 10 µM glutamate similar to the control (Figure 5a–d).

### 3.7 Activation of RAGE leads to release of glutamate from astrocytes

**Figure 6:**
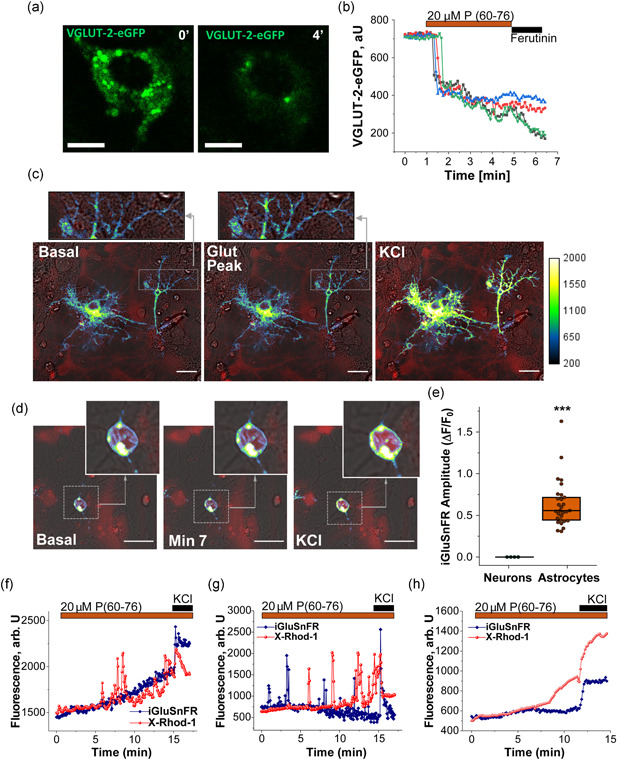
P (60–76)‐mediated RAGE activation leads to vesicular glutamate release mainly from astrocytes. (a, b) Primary cocultures of neurons and astrocytes were transduced with the green fluorescent VGLUT‐2–EGFP construct, targeted to the vesicular glutamate transporter VGLUT‐2 present in synaptic vesicles, specifically in astrocytes. Decrease in the fluorescence (a, b) indicates fusion of the synaptic vesicles after incubation with the RAGE peptide (60–76) and full depletion after ferutinin 30 μM treatment. Scale bar = 10 μm. (c–g) Primary cocultures were transfected with the intensity‐based iGluSnFR probe to monitor glutamate release and coloaded with X‐Rhod‐1 (red fluorescence) to visualize calcium. Representative images of (c) transfected astrocytes and (d) neurons at different points of the experiment. iGluSnFR fluorescence intensity (au) was color‐coded for clarity, and calibration bar is shown. Scale bar = 20 μm. (e) Amplitude of iGluSnFR signal in neurons and astrocytes. The amplitude of 29 glutamate peaks found in the astrocytes in response to the peptide—no peaks were found in the neurons. Mann‐Whitney test, ***p < .00001. (f, g) Representative traces showing glutamate release (indicated by increase in iGluSnFR fluorescence) in astrocytes and calcium signals (X‐Rhod‐1) in the neighboring neurons. (h) Representative traces showing glutamate release and calcium signals in the same neuron. Fifty millimolar KCl was applied at the end of the experiments as a positive control to depolarize the cells and induce glutamate release. RAGE, receptor for advanced glycation end products; VGLUT‐2, vesicular glutamate transporter

To specifically prove the vesicular glutamate release induced by RAGE activation with the peptide, we used two different approaches. We first transduced the cells with the construct EGFP–VGLUT2 (vesicular glutamate transporter; NM053427) targeted to glutamatergic vesicles specifically in astrocytes (P. Angelova et al., 2018; Bezzi et al., 2004) since astrocytes were the cells where membrane‐located RAGE was more abundant in our cultures (Figure 2). Application of the peptide (60–76) induced after a short delay (5–15 s) the fusion of ~35% of the glutamate vesicles in astrocytes (Figure 6a,b). Subsequent addition of the calcium ionophore ferutinin (30 µM); A. Abramov & Duchen, 2003; Zamaraeva et al., 1997) led to the fusion of the rest of EGFP‐VGLUT2 vesicles in astrocytes. This strongly suggests that activation of RAGE leads to exocytosis/endocytosis of glutamate from VGLUT vesicles in astrocytes.

To prove that the fusion of VGLUT2 vesicles in astrocytes induced by RAGE activation leads to the release of glutamate, neurons and astrocytes were transfected with the intensity‐based glutamate sensor iGluSnFR and coloaded with the calcium indicator X‐rhod‐1 before the experiments. Activation of RAGE in astrocytes by 20 µM peptide (60–76) induced peak‐like (Figure 6c,e,g) or slow elevation (Figure 6c,e,f) of iGluSnFR intensity that confirms the release of glutamate by these cells (see summary in Figure 6e). Importantly, the glutamate release in astrocytes led to calcium signals in the neighboring neurons, as indicated by the increase in X‐Rhod‐1 fluorescence in these cells (Figure 6d–h). It should be noted that the application of 20 µM peptide (60–76) did not induce any immediate changes in iGluSnFR intensity in neurons (Figure 6d–g). However, in some neurons showing calcium signals, a slow parallel increase in iGluSnFR fluorescence could be noted, which could also contribute to the glutamate release. Thus, the activation of RAGE by peptide (60–76) induces glutamate release from VGLUT2 vesicles in astrocytes, which stimulates calcium signals in neurons.

## 4 DISCUSSION

Despite the numerous agonists, which are by now described for RAGE, the name of this receptor is associated with their binding to advanced glycation end products described first (Neeper et al., 1992). Although the mechanism of activation of RAGE is described in a number of studies, one of the major difficulties in the interpretation of these results is linked to the multiple targets and high toxicity of RAGE antagonists. Thus, β‐amyloid could act through RAGE but could also be nonspecifically membrane‐active even in very low concentrations (A. Abramov et al., 2003; Narayan et al., 2014). Considering this, a specific activator for RAGE could help to investigate the mechanism of RAGE activation and, potentially, may help to understand the role of this receptor in physiology and pathology. Importantly, in some experiments, activation of RAGE led to neuronal network‐driven activity resulted in synchronous calcium signal (Figure 1a), which suggests that this mechanism can trigger more global signals in the brain.

Here, we show a specific activation of RAGE by a synthetic peptide from its V‐domain, which binds to RAGE present on the surface of astrocytes (and to lesser degree neurons). This leads to the release of glutamate preferentially from astrocytes (from VGLUT2 containing vesicles), but not from neurons. The release of glutamate stimulates NMDA and AMPA receptors in neurons and induces calcium signals in these cells. Importantly, glutamate release by exocytosis of synaptic vesicles typically occurs in the nerve terminals from neurons, while its reuptake succeeds by neurons and glial cells (Zhou & Danbolt, 2014). However, the fast (millisecond range) release of glutamate from astrocytes has been previously shown for VGLUT2 vesicles (Bezzi et al., 2004; Cali et al., 2008). The RAGE‐induced glutamate release produced only a short and sporadic calcium signal (Figure 1a), which is different from the observed after the stimulation of neurons with low or high (excitotoxic) concentrations of glutamate (A. Y. Abramov & Duchen, 2010; Maiolino et al., 2019) or by synchronic release of glutamate to synapses in neurons (Kovac et al., 2012). This RAGE‐induced glutamate release and calcium signal may play an important role in the physiology of CNS because overactivation of RAGE leads to inhibitory/excitatory imbalance, which can be translated to schizophrenia patients (Dwir et al., 2019). RAGE‐deficient mice showed a reduction in seizure activity in a kainate‐induced epilepsy model, which also confirms the involvement of RAGE in glutamate signaling (Iori et al., 2013). Importantly, RAGE‐induced release of S100B from astrocytes enhances kainate‐induced gamma oscillations in vivo (Sakatani et al., 2008), which also suggests the importance of this mechanism in glial‐neuron communication. Activation of the reactive oxygen production and changes in redox regulation in response to RAGE activation in neurons also potentially can be triggered by glutamate receptors.

Neuron‐glia interaction is vitally important in the physiology of CNS and in the pathogenesis of neurodegeneration (P. R. Angelova & Abramov, 2014). Here, we found a novel pathway for this interaction, which could be important for signal transduction or could also be a potential regulator of the redox balance between cells in the brain.

Using a nontoxic compound for activation of RAGE in various types of cells in the same way we used peptide (60–76) could help us to distinguish the role of RAGE as a physiological or potential prosurvival receptor.

## AUTHOR CONTRIBUTIONS

Anna Kamynina, Noemi Esteras, Dmitry O. Koroev, and Plamena R. Angelova conducted experiments. Anna Kamynina, Noemi Esteras, Plamena R. Angelova, and Andrey Y. Abramov analyzed the data. Anna Kamynina, OMV, Noemi Esteras, and Andrey Y. Abramov wrote the manuscript. Anna Kamynina, OMV, Noemi Esteras, Dmitry O. Koroev, Plamena R. Angelova, and Andrey Y. Abramov corrected the manuscript.

## CONFLICT OF INTERESTS

The authors declare that there are no conflict of interests.

## Data Availability

Data available on request from the authors.
